# Language control in bilinguals: The adaptive control hypothesis

**DOI:** 10.1080/20445911.2013.796377

**Published:** 2013-05-24

**Authors:** David W. Green, Jubin Abutalebi

**Affiliations:** 1 Cognitive, Perceptual and Brain Sciences, Faculty of Brain Sciences, University College London, London, UK; 2 Faculty of Psychology, Vita-Salute San Raffaele University and San Raffaele Scientific Institute, Milan, Italy; 3 Division of Speech and Hearing Sciences, University of Hong Kong, Hong Kong

**Keywords:** Adaptive control hypothesis, Bilingual, Cognitive control, Language control

## Abstract

Speech comprehension and production are governed by control processes. We explore their nature and dynamics in bilingual speakers with a focus on speech production. Prior research indicates that individuals increase cognitive control in order to achieve a desired goal. In the adaptive control hypothesis we propose a stronger hypothesis: Language control processes themselves adapt to the recurrent demands placed on them by the interactional context. Adapting a control process means changing a parameter or parameters about the way it works (its neural capacity or efficiency) or the way it works in concert, or in cascade, with other control processes (e.g., its connectedness). We distinguish eight control processes (goal maintenance, conflict monitoring, interference suppression, salient cue detection, selective response inhibition, task disengagement, task engagement, opportunistic planning). We consider the demands on these processes imposed by three interactional contexts (single language, dual language, and dense code-switching). We predict adaptive changes in the neural regions and circuits associated with specific control processes. A dual-language context, for example, is predicted to lead to the adaptation of a circuit mediating a cascade of control processes that circumvents a control dilemma. Effective test of the adaptive control hypothesis requires behavioural and neuroimaging work that assesses language control in a range of tasks within the same individual.

Specific forms of training such as learning to play the piano (e.g., Bialystok & Depape, 2009) and patterns of upbringing (e.g., Hedden, Ketay, Aron, Markus, & Gabrieli, 2008) shape how individuals perform in nonverbal tasks tapping processes of cognitive (executive) control. Strikingly, the use of more than one language appears to be a further important factor shaping individual performance on such tasks (e.g., Bialystok, Craik, Green, & Gollan, 2009, for a review). Why might this be? There is a two-step argument: Increased cognitive demands associated with language control in bilingual speakers lead to enhanced skills in cognitive control and these enhanced skills are deployed in performing nonverbal tasks tapping such control. We focus here on the first step: the cognitive demands of language control in bilingual speakers.

We specify the language control processes involved in three different real-world interactional contexts (single language, dual language, and dense code-switching). By an interactional context we refer to the recurrent pattern of conversational exchanges within a community of speakers. Our thesis is that the control processes themselves adapt to the demands imposed on them by these different contexts. This thesis entails theoretical work to identify the nature of such adaptations. It also implies that experimental tests take explicit account of the contexts of language use.

On evolutionary grounds, we consider that the processes involved recruit processes involved in the control of action in general (e.g., Stout & Chaminade, 2012). They are recruited then in the speech of monolingual speakers too.

This evolutionary assumption poses a puzzle. Given the vast range of skills that individuals learn why, in principle, might learning to use a further language make a difference to the exercise of cognitive control and so affect the cognitive control of nonverbal tasks? Each nonverbal skill would seem to demand the kinds of control processes relevant to language control: For instance, individuals must monitor the context, maintain the action goal and resist interference from other competing actions that may be triggered by the situational context. Is language control special in some way?

Conceivably there is a difference in the complexity of what is controlled. Many nonverbal activities, though complex (e.g., making a cup of tea), involve stereotyped sequences. Communicative actions involving language are arguably relatively less stereotyped and so more demanding in their planning and execution. However, much everyday discourse involves simple linear syntactic structures that are relatively undemanding in terms of their planning and execution (Frank, Bod, & Christiansen, 2012).

A further difference may be more crucial. In the case of nonverbal actions, there can be alternative ways to achieve a given goal. These alternatives are equifinal and we may execute the one that is available faster perhaps because its planning is simpler given the context. For example, we elect to switch off a kettle with our left rather than the right hand whilst holding a cup in the other. In the case of speech, alternative (congruent) utterance plans are not invariably substitutable. Speakers, whether bilingual or monolingual, may need to select between different ways to conceptualise an event and select between different ways to express this conceptualisation depending on their addressee. For bilingual speakers, alternative utterance plans in different languages are not invariably substitutable as the addressee may not know the other language or there may be social or topic reasons for not using it. We suggest therefore that use of language whether in monolingual or bilingual speakers cannot be equated with the myriad of equifinal nonverbal actions: It is a special kind of action in which congruency is an insufficient criterion for selection. Substantial experimental evidence indicates that in bilingual speakers both languages are active even when only one is being used (see, for reviews, Bialystok et al., 2009; Kroll, Bobb, & Wodniecka, 2006). On this view, selection follows activation of alternative possible candidates for expressing a message. In bilingual speakers, the demand to select an utterance despite “equifinality” recurs in a repeated and sustained fashion. Accordingly, we infer that, in principle, language use in bilingual speakers increases the demand on the processes involved in utterance selection over and above those that are imposed on monolingual speakers. If control processes adapt to such demands then this argument provides a basis for expecting possible advantages in the cognitive control of nonverbal tasks though it leaves open the mechanism involved. More critically, it requires us to specify the precise processes that might be subject to adaptation and how the contexts of language use may shape such adaptation. Our goal is to identify a set of language control processes that support conversation in different interactional contexts, articulate the relative demands of these contexts on these processes, and spell out the neural bases of adaptive changes.

Competing representations may extend over the entire speech pipeline from formulating the message, selecting and sequencing relevant lemmas and word forms, to retrieving, and articulating relevant phonemes and monitoring self-produced speech with respect to its predicted acoustic/phonetic form. The targets of language control then may differ in their linguistic level and so the precise locus of control effects will vary (Kroll et al., 2006). We recognise that comprehension processes in bilingual speakers are relevant to the adaptive response. They may tune the system to detect critical features that discriminate one language from another (Krizman, Marian, Shook, Shoe, & Kraus, 2012; Kuipers & Thierry, 2010) and adapt processes that control interference between competing word meanings (e.g., Macizo, Bajo, & Martin, 2010). However, we focus on speech production because the ability to formulate a relevant message is also vital to comprehension. In listening to a speaker we develop a forward model of what they may say and this allows us to act collaboratively. We spell out our proposal in terms of the adaptive control hypothesis in the next part of the paper. In the third part we review and discuss the hypothesis before concluding.

## THE ADAPTIVE CONTROL HYPOTHESIS

We envisage that cognitive control processes select competing representations in working memory as individuals seek to achieve their intended goals. The targets of these control processes differ: they may be verbal or nonverbal representations. In consequence, disruption of the neural linkage between the regions involved in control and the specific target of control can give rise to dissociations in performance despite a set of common control processes.

Prior research indicates that deviations from required performance (e.g., overt errors or delays in responding) trigger control processes that serve to bring behaviour more in line with what is required (e.g., Botvinick, Cohen, & Carter, 2004). So, for example, individuals respond more slowly to the direction of a target arrow when it is flanked by arrows pointing in the opposite direction. However, this conflict effect is reduced when an incongruent stimulus is presented on an immediate subsequent trial. Neuroimaging work implicates a feedback circuit in which signals from a midfrontal neural region (i.e., the anterior cingulate cortex) that detects conflict trigger a response in a left inferior frontal region that serves to suppress interference (e.g., Kerns et al., 2004). We argue for the stronger hypothesis, the adaptive control hypothesis, in which the processes of control themselves adapt to the demands placed upon them. For bilingual speakers the interactional context in which they find themselves drives the adaptive response. Why might control processes adapt? One reason, we suggest, is that there is an interactional cost in not doing so. We look at this cost in a later section.

**Figure 1.**
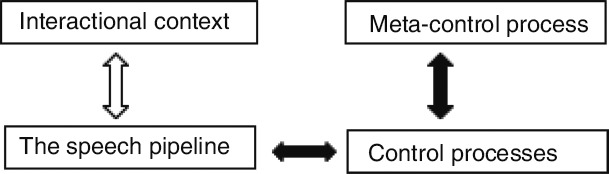
Architecture of the adaptive control hypothesis. Filled arrows depict internal processes of control.

Conceptually then, and also by way of overview, we distinguish the interactional context, the speech pipeline (that is, the conceptual-affective-linguistic-sensorimotor representations entrained in the production and comprehension of speech); the processes that control these representations in working memory to ensure the communicative goal and the process that sets the parameters of these control processes—the meta-process (see Figure 1 for a depiction). It is these parameters that are subject to adaptation and mediate changes in skill. Neurally, adaptation may be achieved in different ways: through a change in structural resources or capacity (e.g., grey matter density), through a change in regional efficiency (e.g., through tuning neuronal populations or changing the responsiveness of neuronal populations) or through a change in the connectivity of the network (e.g., white matter connectivity). Such parameters then capture the persistence or flexibility of control, the efficiency of transmission across the network, and the coordination of different control processes.

In the following three subsections, we describe the interactional contexts, identify a set of control processes used in these contexts, and then consider how the demand on these control processes varies as a function of the interactional context.

### Three interactional contexts

We consider three interactional contexts (three different recurrent patterns of conversational exchange) as a way to contrast demands on control processes. We use these contexts, rather than more specialised contexts (e.g., simultaneous translation, air-traffic control) because these contexts reflect the everyday conversational use of language.

(1) A single-language context in which one language is used in one environment and the other in a second distinct environment. For example, a nonnative language may be spoken exclusively in the work environment with colleagues, whereas the native language is used exclusively with family members at home. In such a context there is no frequent switching between languages;
(2) A dual-language context in which both languages are used but typically with different speakers. Switching between languages may occur within a conversation but not within an utterance.
(3) A dense code-switching context in which speakers routinely interleave their languages in the course of a single utterance and adapt words from one of their languages in the context of the other. For example, in French-Alsatian code-switched speech, a speaker may adapt French verbs through the addition of a German particle (-ieren) as in “choisieren” from the French “choisir” rather than switch to the German word for chose, “wählen” (Edwards & Gardner-Chloros, 2007). In English-Tagalog code-switched speech too there is morphosyntactic adaptation as in: “Wala akong cash pang grocery ngayon, if you want, bukas na lang, ipagdadrive pa kita! [English translation: *I do not have cash for grocery today, if you want, tomorrow, I will even drive you there!*]. The phrase “ipagdadrive” [*I will even drive*] is a code-switched stretch of speech comprising a personal pronoun, auxiliary, modifier, and verb.

### Eight cognitive control processes

Previous proposals have captured broad differences in classes of control operations. In reviewing accounts of research on bilingual advantages in cognitive control tasks, Hilchey and Klein (2011) distinguished accounts that emphasise control processes that maintain a task goal and monitor for conflict over those that emphasise the need to inhibit competing representations. Of course, the processes of goal maintenance, conflict monitoring, and interference suppression are all needed for implementing a given task such as speaking in one language rather than another. One way to recognise the interplay of these processes is to distinguish proactive control processes that establish a task goal from reactive control processes that limit interference with it (Braver, 2012). This contrast is implicit in the inhibitory control model (Green, 1998). In this model, the selection of a language for speaking requires the activation of the task schema for that language (a language task schema). Its activation is increased because the intended language is specified in the conceptual representation. Schema selection arises through competition with the schemas for other languages and also, potentially, through the reactive inhibition of representations that trigger selection of these competing task schemas (see Morales, Gomez-Ariza, & Bajo, in press, for explicit recognition of this contrast and a novel experimental test of its implications for bilingual performance). Our basic supposition here is that the language task schemas are in a competitive relationship in the single language and dual language contexts but are in co-operative relationship in the dense code-switching context. How the schemas are coordinated affect the various control processes that are associated with them.

In order to articulate the dynamics of control, we refine the decomposition proposed by Miyake et al. (2000). These authors distinguished between maintaining and updating information such as task goals; inhibiting competing representations and switching between tasks.

We consider conversation in a dual-language context as a way to achieve a plausible decomposition or fractionation of control processes and assess the likely interdependence of these control processes. The left column of Table 1 lists the eight control processes.

**TABLE 1:** Demands on language control processes in bilingual speakers as a function of the interactional context relative to demands on the processes in monolingual speakers in a monolingual context

	*Interactional contexts*		
*Control processes*	*Single language*	*Dual language*	*Dense code-switching*
Goal maintenance	+	**+**	=
Interference control: conflict monitoring and interference suppression	+	**+**	=
Salient cue detection	=	+	=
Selective response inhibition	=	+	=
Task disengagement	=	+	=
Task engagement	=	+	=
Opportunistic planning	=	=	+

+indicates the context increases the demand on that control process (more so if bolded); =indicates that the context is neutral in its effects. Please see main text for explanation of the control processes.

A speaker must establish and maintain a task goal such as speaking in one language rather than another. We refer to this process as goal maintenance in Table 1. A face-to-face conversation is inherently multimodal and so a variety of cues such as the voice, face, and gestures of the addressee may support goal maintenance. However, other cues in the immediate environment such as the voices of other speakers talking in the other language may activate the goal of speaking in the other language. Maintaining the current goal requires processes that control interference.

We identify two such control processes in line with Kerns et al. (2004): one that monitors for conflict (conflict monitoring) and a process that suppresses interference (interference suppression). These two top-down processes of control are needed to sustain the current language goal. We take inhibitory processes to be central to the control of interference for neurocomputational reasons: to speed up the time course for ensuring maintenance of the current goal, or for the efficient selection of a new goal and to avoid catastrophic increases in activation (see Abutalebi & Green, 2007). The precise locus of suppression in bilingual speakers will depend upon the source of the interference. For example, it may be at the level of the language task schema itself or at the level of particular lexical or syntactic competitors (e.g., Green, 1986, 1998; Kroll et al., 2006). We also leave open the precise mechanism of suppression. It may be one that directly inhibits the competing representation. Alternatively, it may be one in which the target representation and competing representation are interconnected via mutual inhibitory links and so increasing the activation of the target leads to suppression of the competitor indirectly.

The detection of salient cues is integral to successful conversation and in a dual-language context a salient cue such as the arrival of a new addressee may require the speaker to switch to their other language because they typically use that language with that addressee. We therefore consider salient cue detection to be a control process. Experimentally, research on cue detection is tested in a go/no-go paradigm. But such a process may also be recruited to inhibit a prepotent ongoing response so as to allow a more task relevant response (e.g., Forstmann et al., 2008). We term this latter process “selective response inhibition”. Here, it serves to stop the person continuing to speak in the current language and triggers disengagement from it (task disengagement). Switching between languages requires an individual both to disengage from the prior task and to engage with the new one (task engagement). The speed of switching from one task to another depends on this disengagement-engagement cycle. Selective response inhibition might reasonably be viewed as an instance of interference suppression rather than as independent control process but it is triggered by the need to change the task goal and so we retain it as a special case of interference suppression.

Switching between tasks also involves conflict monitoring and interference suppression as a previously active task schema must be suppressed and a new one activated. We consider task disengagement and task engagement as distinct processes because a change of task has wide ramifications in terms of how other processes are configured. Speaking in English as opposed to Mandarin, for example, requires a shift in vocabulary, syntax, and prosodic patterns. In fact, experimental work looking at the sequential behavioural effects of interference and task switching suggests that these are best captured by distinct conflict-control circuits (Brown, Reynolds, & Braver, 2007). Neuroimaging data also implicate distinct neural regions in task switching and interference control (e.g., Cools & D'Esposito, 2011).

Table 1 lists one further control process that we term “opportunistic planning”. By this we mean making use of whatever comes most readily to hand in order to achieve a goal. Speakers in general may plan their speech opportunistically but we have a specific sense in mind. In the case of bilingual speakers, we mean adapting the words of one language to fit into the syntactic frame of another as in the preceding examples. Less proficient speakers of a language may also plan their utterances opportunistically by, for example, recruiting gesture to convey meaning, but the control demand in their case reflects the absence of suitable linguistic means rather than the flexible use of available means.

### Interactional contexts and demands on control processes

In Table 1 we indicate whether the specific interactional context increases demand on a control processes (+) or is unlikely to affect it (=) compared to the demand experienced by a monolingual speaker in a monolingual interactional context. This assessment is also a judgement about the differential effect of the interactional contexts on control processes within bilingual speakers.

How might the different interactional contexts affect the demand on these control processes? If both languages are active and compete for selection, then demand on processes associated with goal maintenance, conflict monitoring, and interference suppression may be high across all contexts. The contexts differ though in the explicit presence of other languages. Both languages are present in the dual-language and dense code-switching contexts but not in the single-language context. The contexts also differ in how interference may be resolved. In the single-language and dual-language contexts interference must be resolved so as to avoid switching into the other language. The language task schemas are in a competitive relationship. By contrast, in the dense code-switching context, opportunistic planning can make use of alternative forms of expression that would be competing in another context. The language task schemas are in a co-operative relationship. This does not mean that code-switched speech is not cognitively demanding. Our thinking is that dual-language activation in a dense code-switching context creates opportunities for morphosyntactic integration. However, though such a context may circumvent the need for the strong suppression of alternatives, it imposes a demand on the fine temporal control of morphosyntactic processes. As a first approximation then, we suggest that the demand on the control processes of goal maintenance, conflict monitoring, and interference suppression, is highest in the dual-language context. By contrast demand on opportunistic planning is highest in the dense code-switching context.

In Table 1 we also indicate a differential demand on the process of salient cue detection and on the subsequent cascade of control processes that concern selective response inhibition, and task disengagement and engagement. The basis of this claim lies in the recurrent demand for speakers in a dual-language context to circumvent a control dilemma.

By way of illustration of this dilemma, consider a conversation in a single-language context. Imagine Farsi-English speakers in Iran who must communicate in their second language (English) because their visiting colleague only speaks English. In order to do so they must establish and stabilise a particular control state in which, for instance, any competing linguistic representations from Farsi are suppressed (i.e., the language task schema for English is dominant). However, a control state in which current goals and actions associated with them are stabilised, that is, a state resistant to change or interference, is at odds with a state that favours flexibility in switching to new goals in response to new inputs. In this scenario, it is the arrival of a colleague who speaks Farsi but not English. This control dilemma, in which effective suppression limits flexibility to respond to new cues, is not a recurrent dilemma in the single-language context but it is one in the dual-language context. The dilemma can be circumvented by an independent control process that we have already identified: salient cue detection. Such a process can then trigger processes (selective response inhibition, task disengagement, and task engagement) that leads to a fluent switch in language. We conjecture therefore that this cascade of processes will be subject to adaptation in the dual-language context.

### What drives the system to adapt its control processes?

The prototypical use of language is conversation and conversations are joint actions in which the participants seek to minimise joint effort in achieving a shared situation model (e.g., Clark, 1996). Taking this perspective as our point of departure, we consider what we term the “interactional cost” as a factor that motivates adaptive changes in control processes.

In a single-language context, a conversation in which the speaker repeatedly switched into their native language would disrupt the conversation. Initially, addressees might be sympathetic and attempt to complete utterances for the speaker. Failure to control language use, together with low proficiency in the second language, would also preclude the speaker contributing effectively when the other person speaks. They would fail to develop a suitable forward model to complete an utterance where necessary. The bilingual speaker would fail to ensure that the joint effort involved in the conversation is no greater than necessary in order to build a shared situation model. Repeated conversations on these lines put the bilingual's employment at risk. Nonnative speakers need to change in two ways. They need to increase their second-language proficiency. But in order to increase proficiency in the second language, given that both languages are active, the speaker needs to adapt the processes that control interference especially when using the second language, otherwise there is a computational paradox: An increase in second-language proficiency yields a concomitant increase in interference that reduces fluency. Interactional cost imposes a demand to adapt the control processes of goal maintenance, conflict monitoring, and interference suppression.

In the dual-language context the demands on control processes are more complex and, so too, is the adaptive response. To reduce interactional cost, speakers must sustain attention to the current language goal and suppress interference but be in a position to switch languages on detection of an addressee with whom they converse in their other language. Reducing the interaction cost specifically requires that they manage the control dilemma that reducing interference also reduces sensitivity to relevant external cues.

Finally, in the dense code-switching context we have supposed that a key demand is on opportunistic planning. But where is the interaction cost that drives the adaptive response? Consider a speaker who does not engage in dense code-switching in a community of speakers who do. Such a speaker clearly marks themselves out as an outsider. But their behaviour also imposes a cost on those they talk to because it increases the demand on such speakers to avoid code switching (on grounds of reciprocity). Since such speakers are putatively less practised in controlling interference this increases joint effort and so imposes an interaction cost. Over time, such additional cost may lead speakers in that community to disengage from conversation with the outsider.

In the previous three subsections we have described the basis of the adaptive control hypothesis. We turn now to consider some predictions that might be made in terms of overt behaviour and the neural regions that mediate cognitive control.

### Testing the adaptive control hypothesis

A basic prediction of the hypothesis is that speakers in the three different interactional contexts will show different patterns of adaptive response. All else being equal, the hypothesis is refuted if the interactional context proves irrelevant. Indeed, one might argue that finding any evidence for the effects of the interactional context on any of the control processes would be highly surprising. After all, the need to switch between languages is present in all contexts—only the details vary. The detection of a salient language cue may show little or no variation because detection of salient cues is critical to survival whatever the interactional context. Opportunistic planning may also be intrinsic to everyday conversational practice. For example, in casual speech, speakers make use of previously primed phrases rather than formulate them anew precisely because they are readily to hand.

On the other hand, the core premise that the brain adapts to demands might also make the hypothesis irrefutable because all speakers adjust their behaviour during an experiment to the specific control demands it imposes. However if, as the hypothesis envisages, recurrent control demands lead to specific adaptive changes, then the control states they mediate should be triggered more easily by relevant experimental conditions. In other words, any learning effects during the experiment should be observed earlier for speakers from an interactional context that is best suited to the experimental conditions. In fact, under optimal conditions, the relevant control state should be triggered immediately.

We also acknowledge the issue of circular causation. Interactional context may shape adaptive response but individual differences (in predispositions and genetic make-up) surely constrain such effects. We consider this issue in more detail in the later subsection on individual differences. For now, we assume that individual variation in executive control processes and, in sociality, is normally distributed in speakers in the three interactional contexts. In consequence, adaptive effects of these contexts should be observable. We consider behavioural predictions first of all.

### Behavioural predictions

We expect that control processes are predictive of performance in conversational, dialogic tasks where joint effort must be minimised (see Festman, 2012, and Pivneva, Palmer, & Titone, 2012, for novel research examining the link between executive control tasks and conversational performance in bilingual speakers). Suppose individuals have to describe a depicted event to an addressee. In one experimental condition they are free to switch between languages at will. In another they are required to switch on cue into one of their languages or the other. For speakers from a dense code-switching context, fluent performance will be associated with the freedom to use either language, whereas an imperative cue requiring them to restrict their utterances to a single language will impair performance. By contrast, baseline fluency will be relatively greater for those bilinguals from the single-language and dual-language contexts when only one language is required. For speakers from these two contexts, the adaptive control hypothesis makes further predictions. A cue to switch languages should trigger a set of processes where the cascade is more integrated for bilinguals in the dual-language context compared to those from a single-language context. Two effects are predicted. The transitory cognitive load imposed in response to the imperative cue might occur earlier for speakers from a dual-language context and so affect their current speech precisely because the cue sets processes in train to switch language more rapidly. However, as a corollary, once the switch in language has occurred, hesitation and speaking rate in such speakers should return to baseline more rapidly.

The basis of these predictions lies in how the language tasks schemas are coordinated in the different contexts and in the planning processes of speech. In a dense code-switching context speakers establish a cooperative relationship between the schemas. This permits opportunistic planning in which the speech plan reflects the dynamic accessibility of words and constructions regardless of their language membership and incorporates items contingent solely on them meeting current syntactic constraints. In such circumstances there is no basis for predicting a switch cost. The same possibility exist for speakers from the other contexts instructed to switch at will but they have to overcome their default coordination in which the language tasks schemas compete to control output and so routinely restrict access to the speech plan to items and constructions from just one language.

Whereas switching language during sentence production may incur no overt cost for speakers from a dense code-switching context, switching language in response to an imperative cue to name a picture will continue to incur a cost even for speakers in a dual language context adept at switching between languages because in such circumstances speakers must continue to establish a competitive relationship between the language schemas.

With respect to experimental tasks that tap specific component control processes adaptive effects should be evident in the analysis of reaction time distributions for conflict tasks such the colour-word Stroop task. Interference effects can be plotted as a function of response speed and these plots show that interference effects decrease for slower responses and more so for individuals who are more proficient in inhibition. Our analysis leads to the prediction that bilingual speakers in the dual-language context will be the more proficient in inhibition than those in the single-language or dense code-switching contexts.

A more critical prediction concerns how speakers adapt to the control dilemma in which a suppressive state limits flexibility to respond to a cue indicating a new task. Experimentally we can examine the relationship between response to an interference trial and a response to an immediately following trial involving a language switch. We can attempt to maximise sensitivity by ensuring that the cue signalling a different language coincides with a property that must be suppressed (cf. Goschke & Dreisbach, 2008). In a colour-word Stroop task, the written word is the suppressed property and so a printed cue on a following (neutral) trial that signals a change in language should be less readily detected. For speakers in the single-language and dense code-switching contexts reduced verbal interference on the interference trial will be associated with increased language switching costs. By contrast, if speakers in the dual-language context have adapted to circumvent the control dilemma then these two effects should dissociate. We have proposed a test using a visual Stroop task yet the nature of any adaptive response may be specific to the multimodal nature of the typical conversational exchanges. A cross-modal Stroop task is perhaps more pertinent. Here the auditory input is the suppressed dimension and the salient cue is an auditory one (e.g., in the language to be switched into). Indeed, testing the specificity of the cue (to known voices vs. the voices of others) provides a way to explore the precise tuning of the process of salient cue detection.

### Neuroimaging predictions

**Figure 2.**
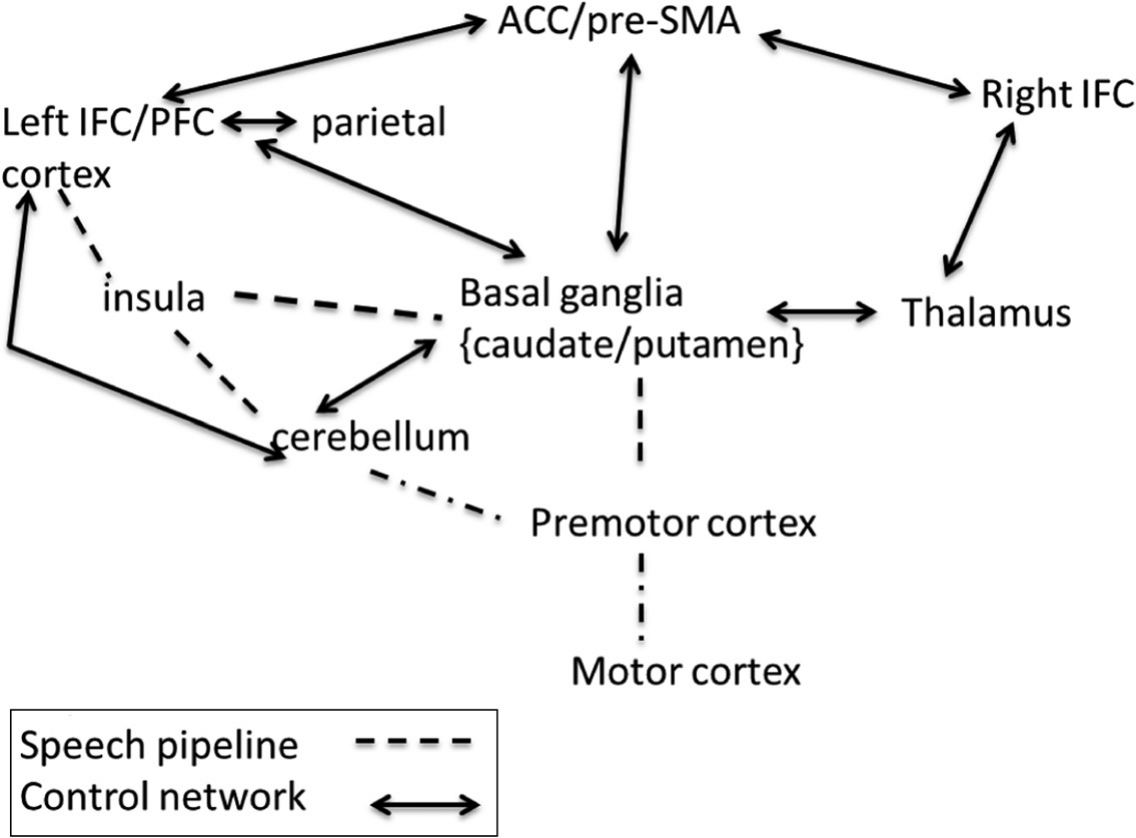
Simplified language control network and speech production regions (see main text for explanation).

Empirical research (e.g., Luk, Bialystok, Craik, & Grady, 2011) that has established a widespread difference in the brains of adult monolingual and bilingual speakers encourages the search for the nature and origins of specific adaptive changes. We consider predictions that relate specific control processes to particular neural regions and networks. Figure 2 provides a schematic description of the neural structures and their connections that we associate with language control processes. In the figure the networks involved in language control are indicated with bidirectional continuous arrows, whereas those indicating part of the speech pipeline are indicated with dashed lines.

For the speech pipeline we reference regions based on the analysis of verbal fluency data (Eickhoff, Heim, Zilles, & Amunts, 2009). We treat these as representative of regions active in self-generated speech and a subset of those active in conversational speech. Unrepresented in the figure is the monitoring of speech output that involves anterior and posterior regions of the temporal cortex. Also unrepresented are input regions to the left cortex that contribute to the conceptual content to be expressed. In previous papers (e.g., Abutalebi & Green, 2007, 2008), we reviewed work showing that both cortical and subcortical structures are involved in language control and language switching (see Luk, Green, Abutalebi, & Grady, 2012, for a recent meta-analysis). In this work we identified the anterior cingulate cortex (ACC) and the presupplementary motor area (pre-SMA) with conflict monitoring and acknowledged a role of the pre-SMA in initiating speech in language switching (see Luk et al., 2012, for further discussion). We associated the control of interference with left prefrontal and inferior cortex, parietal cortices with the maintenance of task representations, and one subcortical structure (the caudate) in the basal ganglia with the switching between languages (Abutalebi et al., 2013). On one proposal, basal ganglia circuits, more generally, serve to control or gate access between prefrontal cortex and posterior cortical regions that represent task information (Cools & D'Esposito, 2011; see also Frank, 2011). We extend the networks involved in language control in Figure 2.

In Figure 2 we include a direct link between frontal cortex and the right cerebellum as a further circuit involved in language control (Fabbro, Moretti, & Bava, 2000). Neuropsychological data and functional data indicate that damage to the right cerebellum suppresses activation in left frontal cortex and elicits aphasic symptoms typical of those shown by frontal lobe patients. Reperfusion of the cerebellum reduces such symptoms (Marien, Engelborghs, Fabbro, & De Deyn, 2001). Neuropsychological data implicate cerebellar structures specifically in the control of morphosyntax such that their damage leads to substitution of inappropriate bound morphemes (Silveri, Leggio, & Molinari, 1994). We therefore consider that it will play a critical role for speakers in dense code-switching contexts.

We also include a circuit involved in the detection of salient cues (salient cue detection). This circuit involves regions in right inferior frontal cortex (e.g., Aron, Behrens, Smith, Frank, & Poldrack, 2007) and the thalamus. The thalamus directly accesses regions of the basal ganglia such as the caudate and putamen (Smith, Surmeier, Redgrave, & Kimura, 2011). We note that left subcortical regions such as the caudate seem more involved in the control of verbal interference (e.g., Abutalebi et al, 2008; Ali, Green, Kherif, Devlin, & Price, 2010). Finally, we suppose reciprocal connections between basal ganglia structures and the cerebellum (Bostan, Dum, & Strick, 2010).

Adaptive effects should be expressed in the neural regions and circuits that mediate the control demands in each context. For example, in order to implement the cascade of control processes required in the dual-language context, the hypothesis predicts changes in the network comprising cortical, thalamic, and basal ganglia regions. In addition, there is a demand on frontal structures linked to conflict monitoring and interference suppression (e.g., Kerns et al., 2004) and to parietal regions (and frontal regions) associated with task changes. By contrast, the hypothesis predicts an adaptation in speakers in dense code-switching contexts involving the connectivity of right cerebellar and left inferior frontal regions. In the single-language context, the demand is to ensure efficient suppression of the nontarget language over extended periods of time. To the extent this is successful, there is no additional demand on subcortical structures associated with language switching.

Adaptive effects may also be revealed by the patterns of neural activation as speakers from the different contexts perform the same task, by the distinct correlations of behavioural performance with measures of structure or connectivity and in neuropharmacological assessments. We present an illustrative prediction for each type in the following paragraphs.

Recent work has distinguished different inhibitory mechanisms in the control of language: those involved in globally suppressing language representations (relevant perhaps when a language is used over an extended period of time) and those involved in more local suppression when participants switch on cue between two languages. One study (Guo, Liu, Misra, & Kroll, 2011) contrasted a condition in which picture naming was blocked by language and one where participants switched between their two languages. Guo et al. (2011) observed that the ACC (and supplementary motor area) was more active during language switching (consistent with local inhibition) and the dorsal left frontal cortex and parietal cortex were more active during language blocking (consistent with global inhibition). Indeed in a context where just one language is used for naming, frontal regions but not subcortical regions, show increased activation in bilingual relative to monolingual speakers and such activation is linked to the control of interference (Parker Jones et al., 2012). One interpretation is that frontal regions in such a context select the target name from an activated set of competing names at an early stage in the production process (i.e., before release of the utterance plan). By contrast, subcortical regions play a more critical role during switching consistent with a later selection of the target name (Green, 2011). The adaptive control hypothesis predicts that the precise pattern of blocked naming effects will depend on the interaction context. The difference in neural response between blocked naming and language switching should be greater for those in the single-language interaction context who are unused to language switching compared to those in the dual-language context. Left caudate activation should show a marked increase reflecting the increased demand on processes involving late selection. Speakers from a dense code-switching context may show a distinct profile in which they use the cerebellar-frontal circuit to mediate language switching.

The adaptive control hypothesis predicts that the interactional context differentially affects processes that circumvent the control dilemma in which a suppressive state, induced to control interference, makes the system less response to a cue signalling a change in task. How might this prediction be tested? Prior neuroimaging research (Luk, Anderson, Craik, Grady, & Bialystok, 2010) has combined an interference suppression task with a go/no-go task, but we need a paradigm in which individuals respond to a salient cue despite a suppressive control state. We can use the experimental task described previously that combines a verbal interference task with language switching. A common pattern across the three contexts should be found for the interference trials: increased activation in left inferior frontal regions predictive of reduced interference (e.g., Ali et al., 2010; Parker-Jones et al., 2012). Of specific interest are the data in which a language switch trial follows an interference trial. On these language switching trials, there should be distinct effects of interactional context. The required cascade of control processes should be better synchronised for speakers from the dual-language context compared to speakers from the single-language and dense code-switching contexts. One possible correlate is a stronger coupling of activation in right inferior, thalamic and basal ganglia regions and frontal-parietal regions. Probing the cascade more precisely requires studies that relate behavioural indices, from tasks that tap specific control processes, to the neural response.

### Individual differences

We have illustrated how the three interactional contexts lead to adaptive changes in control processes. The proximal cause of adaptive change is a person's engagement with the recurrent forms of conversational exchange in that context. It follows that we can recast our proposal at the level of the individual speaker. We can ask about the extent to which they engage in the type of exchange suited to a given context. We can also ask about the extent to which they experience different interactional contexts, that is their individual behavioural ecologies. We consider this question first of all.

Our three interactional contexts are defined by a single type of exchange but, in principle, speakers may experience all three contexts and may experience these contexts to different extents. We can envisage a space of possible speakers characterised by the distribution of the types of conversational exchange in which they engage. For example, a speaker may have a preponderance of single-language context exchanges, some dual-language context exchanges, and no dense code-switching exchanges. Another may have a preponderance of dense code-switching exchanges, some single-language context exchanges, and no dual-language context exchanges. According to the adaptive control hypothesis, the precise pattern of adaptive changes to the control processes and their neural basis will reflect the actual pattern of exchanges. Such variety in the types of exchange is not just a theoretical possibility. At least in terms of single-language versus dual-language exchanges it fits the results of an interesting questionnaire-based study of language use in Spanish–Catalan bilingual university students (Rodriguez-Fornells, Krämer, Lorenzo-Seva, Festman, & Münte, 2012). In this study, although two-thirds of the sample used both Catalan and Spanish at university, two-thirds used either one or the other language at home. For simplicity's sake, though we endorse our recasting, we will continue to refer to the interactional contexts as these define the specifics of the adaptive response.

We turn now to other factors that may constrain how individuals respond in a given interactional context. We argued that speakers adopt conversational practices suited to that interactional context because there is an interaction cost in not doing so. One constraint on their ability to avoid such a cost is their proficiency in the two languages. The relationship between proficiency and specific adaptive changes as a function of interactional context is unlikely to be straightforward and we have implicitly assumed that our speakers are highly proficient in both languages and so examined adaptive changes under conditions of relatively stability from a proficiency point of view. We simply note here that for speakers in single- and dual-language contexts an increase in proficiency is most likely associated with increased skill in the control of interference. The same may only be true for those in dense code-switching contexts until they can begin to use their knowledge of the two languages opportunistically.

A different constraint is that individuals may vary in their sensitivity to this interaction cost—perhaps because they differ in sensitivity to social cues in general. In consequence they may not engage in the conversational practices that impose the recurrent demands needed to entrain adaptive changes. Accordingly, a corollary of the hypothesis is that sensitivity to interaction cost will predict the extent to which individuals engage in exchanges typical of that interaction context. Testing this prediction requires an analysis of individual conversational practice. A further issue is the quantitative relationship between the number of recurrent exchanges and adaptive changes. We presume the relationship is nonlinear and reflects the typical relationship between practice and performance.

Sensitivity to interaction costs is not the only factor that may constrain adaptive change. Individuals vary in their capacity for cognitive control. Scores on tests of executive function are known to predict cross-language intrusion errors in which words from a person's first language intrude on their speech in their second language (e.g., Festman, 2012; Rodriguez-Fornells et al., 2012). In the single and dual-language contexts, the integrity of left frontal structures will predict cross-language intrusion errors. Potentially, cross-language intrusions can be used opportunistically in dense code-switching contexts. If such opportunistic use requires the integrity of the right cerebellar-left frontal circuit, then indices of its integrity (e.g., regional variations in grey matter density or white matter connectivity) will predict the extent to which a speaker engages in dense code-switching and their facility and perhaps pleasure in doing so.

Examining the role of individual differences in adaptive response is a discovery procedure. It provides a way to identify different circuits that may mediate the same control process. Suppose, by way of example, that, as predicted, the right cerebellar-left frontal circuit showed adaptive changes in speakers in a dense code-switching context. Impairment to this circuit should then preclude dense code-switching if that circuit was necessary. The identification of speakers with such impairment whose code switching was normal from a behavioural point of view immediately implies that there must be at least one other circuit that can fulfil the function. Individual differences therefore provide a way to explore such degeneracy in control processes (Green, Crinion, & Price, 2006).

Preexisting neuropharmacological differences are also relevant to the adaptive response of the control systems. Individuals with increased dopamine receptors in dorsal striatal regions (the caudate and putamen) stop more quickly in response to a stop signal (a salient cue) and their response profile is associated with increased inhibition-related activation in frontal-striatal pathways (Ghahremani et al., 2012). In turn, dopamine receptor values depend on versions of the COMT gene. Current data suggest that individuals with one allele (Met) are better at tasks that require interference suppression whereas individuals with the other variant (Val) are better at switching to a new task (Cools & D'Esposito, 2010). It would be of great interest to see how the interactional context shapes performance when these genetic differences are taken into account. The adaptive control hypothesis predicts that processes associated with salient cue detection are targets for adaptation. One speculative prediction is that receptor density will change as function of control demands. The adaptive control hypothesis then promotes longitudinal studies charting adaptive changes in bilingual speakers in the light of genetic variation.

## REVIEW AND DISCUSSION

We have proposed the adaptive control hypothesis. We identified interaction cost as a factor that drives control processes to adapt with the precise adaptation shaped by the interactional context. The processes of cognitive control adapt by changing the parameters of their operation including their coordination with other control processes. Speakers in a single-language seek to maintain the current language goal and avoid cross-language intrusions. We associated effective suppression of the nontarget language with left inferior regions. Speakers in a dense code-switching context opportunistically use joint language activation to create novel mixed-language utterances. On the basis of neuropsychological data, and the requirement for fine temporal control in this context, we associated adaptive change with the left frontal and right cerebellar circuit. Speakers in the dual-language context are faced with a control dilemma: A suppressive state that limits interference from the current nontarget language also restricts the speed of response to a cue signalling a change to that language. We proposed that they circumvent this dilemma by linking a region that detects salient cues to those involved in selective response inhibition, task disengagement, and engagement. This circuit includes a right inferior frontal region, the thalamus, and basal ganglia structures. We have provided illustrative behavioural and neuroimaging predictions.

Individual differences are also pertinent to testing the hypothesis. The proximal cause of adaptive change is the recurrent exchanges typical of that context. However, individuals may vary in the extent to which they engage with typical recurrent exchange associated with a given interactional context and over what period of time. Differences in sensitivity to interaction cost, differences in the capacity for cognitive control and differences in circuit neuroanatomy may all constrain engagement and affect the degree of adaptive change. Individuals may also experience different interactional contexts (single language, dual language, dense code-switching) and so the adaptive response will reflect the distribution of exchanges typical of those contexts.

It is possible too that individuals differ in how they respond to control demands. We have supposed, as a first approximation, that goal maintenance, conflict monitoring, and interference suppression will all show adaptive changes in speakers in a dual-language context. But it may be objected that this pattern depends on precisely how individuals manage the relative activation of their languages (i.e., the extent of the competitive relation between the language schemas). Conceivably, careful balancing of the relative activation of the two languages (e.g., through proactive control) may limit the need to control between-language interference (and use reactive inhibition) and so reduce the need to circumvent the control dilemma described earlier. If so, the ability to switch rapidly between languages (involving selective response inhibition and task disengagement and engagement processes) may dissociate from the skilled control of interference and salient cue detection. However, in our view, speaking in one language to the exclusion of another is intrinsically linked to a suppressive state. This state of affairs does not arise in a dense code-switching context. Exploring control states experimentally requires an increased focus on the pattern of performance within individuals on a range of tasks that tap different processes of language control.

Other specific factors may be relevant to particular contexts. For example, in the dense code-switching context, the extent of opportunistic planning will vary with the variety of locally adjusted forms that the speakers use. Speakers will also differ in the novelty of their own code-switched forms (see Wei, 2013). Such variations may be critical to the adaptive response. Other types of code-switching exchange (see Deuchar, Muyksen, & Wang, 2007) may impose somewhat different demands. In the dual-language contexts, speakers may vary in their styles of conversational management. Conversations are multimodal. Face and hand movements are known to facilitate comprehension and to configure the regions involved in language comprehension (e.g., Skipper, Goldin-Meadow, Nusbaum, & Small, 2009). They also allow a nonverbal channel (a smile, a handshake) to signal acknowledgement of a new addressee. In everyday conversational exchanges, such expressions and gestures are integrated with the processes of language switching. An exploration of adaptive changes therefore requires a shift in emphasis to more ecologically valid forms of multimodal exchanges.

### Generalisations to other tasks

In keeping with focus of this paper, we have suggested tests of the adaptive control hypothesis using language-based tasks. The adaptive control hypothesis may also be used to make predictions about the performance on tasks that are not directly tied to language control. At a general level, others have pointed to the relationship between control states required in bilingual speakers and those required for certain types of thinking (e.g., Hommel, Colzato, Fischer, & Christoffels, 2011). The basic idea is that controlling the interference of two language leads to a control state conducive to convergent thinking but inimical to divergent thinking. Using different verbal materials to assess convergent and divergent thinking, Hommel et al. (2011) found that bilinguals were better at convergent thinking and monolinguals at divergent thinking. Such an outcome, we suggest might not hold for speakers from a dense code-switching context. Instead, those adept at dense code-switching might be skilled at a form of mental synthesis (Green, 2011). They might, for example, be faster to envisage how a pair of letters might depict an object (e.g., an umbrella from the letters J and D or a chair from the letters I and N; cf. Pearson, Logie, & Gilhooly, 1999).

The adaptive control hypothesis can also be used to ground more precise predictions about performance of speakers on nonverbal tasks. Existing data point to associations between performance on language switching tasks and nonverbal switching tasks (Prior & Gollan, 2011; Soveri, Rodriguez-Fornells, & Laine, 2011), between intrusion errors in a single-language conversational context and cognitive measures of executive functioning (Festman, 2012) and between measures of language switching and the control of nonverbal interference (Linck, Schwieter, & Sunderman, 2012). The adaptive control hypothesis makes predictions about specific control processes that are contingent on the interactional context or, more generally, the distribution and nature of recurrent exchanges. In that sense it provides a rationale for the detailed characterisation of bilingual speakers if robust and replicable findings are to be obtained. The hypothesis also envisages that it is the pattern of performance on a range of tests that is critical.

One basic prediction is that where a task matches the control demands of that context, bilingual speakers will show more rapid adaptation. Compared with monolingual Spanish speakers, Catalan-Spanish speakers, two-thirds of whom, we may infer from Rodriguez et al. (2012), operate in a dual-language context, displayed better interference suppression in a nonverbal flanker task during early trial blocks (Costa, Hernández, & Sebastian-Gallés, 2008). Speakers from a single-language context, but not those from a dense code-switching context, may also show some benefit. The benefit for speakers in a dual-language context may be linked to the increased efficiency of an anterior cingulate region (see Abutalebi et al., 2011).

In terms of the pattern of performance, superior skill in resolving nonverbal interference should be associated with superior skill in circumventing the control dilemma for those in the dual-language context. Goschke and Dreisbach (2008) showed that individuals were less likely to detect a cue that signalled a different task and required a different response when that cue coincided with a property that had to be ignored in the primary task. The adaptive control hypothesis predicts that relative to monolingual speakers, speakers from the dual-language but not from other bilingual interactional contexts would be more adept at background monitoring and so more frequently detect the cue and make the different response required.

Such associations leave open the precise mechanisms. An advantage may arise because control is exercised by a common mechanism, that is, a common network of neural regions or a common pool of resources. Alternatively, language control may involve a distinct control network that emerges from a more general system mediating action control. Bilingual speakers routinely recruit this specialised language control system to handle nonlinguistic tasks. Monolingual speakers do so too—they also use language to label objects and events and to sequence actions. However, because the language control network is shaped by different demands in the two cases (depending, for example, on the interactional context) different effects arise. In consequence, monolingual and bilingual speakers may display different patterns of relations linking verbal and nonverbal performance (e.g., Blumenfeld & Marian, 2011). Lastly, the network for language control and that for the control of nonlinguistic actions may be jointly active. In this case, differences between bilingual and monolingual speakers may arise because of synchronisation between the two networks with neural response reflecting their joint but distinct operation. Discriminating these alternatives in terms of functional imaging data requires analysis of the causal dynamics of control. Our analyses point to the need for further discriminating tests linked to a detailed profile of the speakers’ use of their languages.

## CONCLUSION

We have concentrated on the suggestion that the interactional context (the typical interactional exchange in those contexts) is important in leading bilingual speakers to adapt their cognitive control processes and to tune the networks of control. We have exemplified the adaptive control hypothesis. Our illustrative predictions may prove false. There may be no systematic behavioural or neural differences as a function of the interactional contexts but we hope to have established that there is value in identifying the demand on control processes and how the precise contexts of use shape their properties, coordination, and cascade. Exploration of the adaptive control hypothesis requires a continued shift to studies that examine the pattern of performance (behavioural and functional) on a range of tasks that tap specific processes of language control. From a practical point of view this shift requires the development of efficient testing protocols. These would also be of clinical relevance. This approach, linked to a detailed characterisation of the behavioural ecology of the bilingual speaker, will play a vital role in the development of neurocomputational models of speech production in such speakers.
